# Health-related quality of life in hypertrophic cardiomyopathy patients with implantable defibrillators

**DOI:** 10.1186/s12955-016-0467-x

**Published:** 2016-04-14

**Authors:** Peter Magnusson, Stellan Mörner, Fredrik Gadler, Jan Karlsson

**Affiliations:** Cardiology Research Unit, Department of Medicine, Karolinska Institutet, Karolinska University Hospital/Solna, Stockholm, SE-171 76 Sweden; Centre for Research and Development, Uppsala University/Region Gävleborg, Gävle, SE-801 87 Sweden; Department of Public Health and Clinical Medicine, Umeå University, Umeå, SE-90187 Sweden; University Health Care Research Center, Faculty of Medicine and Health, Örebro University, SE-70182 Örebro, Sweden

**Keywords:** Adult, Hypertrophic cardiomyopathy, Implantable cardioverter-defibrillator, Quality of life, SF-36

## Abstract

**Background:**

Health-related quality of life (HRQL) in hypertrophic cardiomyopathy (HCM) patients with implantable cardioverter-defibrillators (ICDs) is largely unknown. The aim was to assess HRQL, including comparisons between groups, using the questionnaire SF-36, and compare it to a Swedish age- and sex-matched population.

**Methods and Results:**

Validated data on adult HCM patients with ICDs were used. The SF-36 response rate was 82.5 % and 245 patients (mean age 55.9 years, 70.2 % men) were analyzed using the Mann-Whitney *U*-test, *t*-test, Spearman correlation and effect size calculations. In all SF-36 domains the patients’ score was lower (*p*-value of <0.0001) than norms except for bodily pain. The general health domain showed the highest effect size (0.77) and the impact was more pronounced in the SF-36 physical component summary score (0.62) than the mental component summary score (0.46). Older age was correlated with lower scores on the physical component and higher scores on the mental component. Atrial fibrillation and/or systolic heart failure were associated with worse physical health. HRQL was similar in primary vs secondary prevention cases. Inappropriate ICD shock was associated with worse mental health while appropriate therapy trended toward better mental health.

**Conclusion:**

HCM patients with ICDs suffer from poor HRQL regardless of age, sex, or primary vs secondary prevention indication. Atrial fibrillation and systolic heart failure are determinants of poor physical health. Inappropriate shocks, but not appropriate therapies, are associated with poorer mental health.

## Background

Hypertrophic cardiomyopathy (HCM) in adults is characterized by an abnormal thickening of the left ventricular wall (≥15 mm) that is not explained by other causes, such as hypertension or aortic stenosis [1]. The prevalence is approximately 0.2 % and patients often suffer from dyspnea, chest pain, dizziness, syncope, and palpitations, especially upon exertion [1, 2]. Dyspnea may be aggravated by a left-ventricular outflow obstruction, tachycardia, or systolic heart failure (HF). A history of atrial fibrillation (AF) warrants anticoagulation therapy due to substantial risk of embolization stroke [1]. Ventricular tachyarrhythmias can be life-threatening emergencies. An implantable cardioverter-defibrillator (ICD) is a device implanted in the upper chest with a transvenous lead fixated inside the right ventricle. In the event of a dangerous tachyarrhythmia, the ICD can deliver a shock of energy via the lead directly to the heart in order to convert the arrhythmia back to normal rhythm. The ICD is also able to provide pacing support, when needed, but most importantly, it can protect patients from sudden cardiac death (SCD) [3]. Survivors of an episode of ventricular fibrillation or ventricular tachycardia with hemodynamic compromise are recommended an ICD for secondary prevention of SCD. Primary prevention candidates are selected based on evaluation of established risk factors: unexplained syncope, non-sustained ventricular tachycardia, abnormal exercise response, extreme myocardial thickness (≥30 mm), or a family history of SCD [4, 5]. Recently, a novel risk calculator for HCM has been adopted in European guidelines; however guidelines also consider life-long risk of complications and psychological health [1].

The concept “quality of life” is a cornerstone of various treatment strategies and refers to patient-reported outcome measures which are either generic or specific to a certain disease state. Disease-specific instruments usually focus on perceived health status rather than quality of life, defined as “satisfaction with life.” Generic health-related quality of life (HRQL) instruments include both pure health status and domains reflecting quality of life and are thus more holistic, but they may be less sensitive than disease-specific questionnaires. Only generic measures allow comparisons with the general population. Therefore, we chose the widely used 36-item Short Form (SF-36) Health Survey, a validated, self-reported questionnaire, to assess generic HRQL in HCM patients with ICDs [6, 7]. Several studies have addressed specific concerns with general ICD patients based on various indications, such as risk of anxiety, depression, and worsened well-being [8–10]. In a study of a tertiary center cohort, it appears that prior to the ICD era, HCM patients seem to have a deteriorated HRQL compared to the general population [11]. The HCM subset of ICD patients may differ from other populations in age, burden of symptoms, risk of ICD shocks, family history, comorbidities, and having a longer life expectancy.

The primary aim of this study was to compare the SF-36 health profile in HCM patients with ICDs with age- and sex-matched general Swedish norms for SF-36. The secondary aims were to evaluate the impact on HRQL of appropriate ICD therapy, inappropriate ICD shock, a history of unexplained syncope, systolic HF, AF, device complications requiring surgery, familial SCD, and secondary versus primary indication.

## Methods

We previously reported a longitudinal follow-up of ICD-related complications requiring surgery and those experiencing inappropriate shocks. In the present cross-sectional study we assess HRQL in all living patients [12].

### Data collection, validity, and ethics

In November 2012, we extracted data from the Swedish ICD Registry on patients who had ever had an ICD implanted for a HCM indication. Since its inception in 1995, the Swedish ICD Registry has a validated 98 % coverage of all implants in Sweden. The daily updated online system from the Swedish Tax Authority Census Bureau was used to identify living patients and exclude deceased patients. All living patients were sent an envelope with information about the study, a document to sign their consent of participation in the study, the SF-36 questionnaire in Swedish, and a postage-paid return envelope. A total of three reminders during a period of half a year were sent, followed by phone call to remind those who had still not returned the questionnaire. After the written informed consent was received, we retrieved data from the validated National Patient Register which contains data about diagnoses and hospital admissions and is maintained by The Swedish National Board of Health and Welfare.

Medical records were used to validate registry data and were obtained from the different clinics between December 2012 and April 2014. Data were collected during visits to the hospitals/archives or by receiving copies of medical records by regular mail. Categorization of predefined variables was performed by the investigators (PM and SM).

SF-36 data were manually entered and missing items were addressed by repeated contact with the patient to gain complete answers whenever possible. Quality Metrics (OptumInsight Life Sciences, Inc., RI) provided the SF-36 questionnaire with the license number QM015832. The study was approved by Ethical Review Board in Stockholm (document number 2012/1301-31/3) and complied with current statements in the Declaration of Helsinki.

### Statistical analyses

Descriptive data were expressed as frequencies, percentages, means and standard deviations (SD), and a 95 % confidence interval (CI) was used. Fisher’s exact test and *t*-test were used for comparisons of categorical and continuous variables, respectively. To analyze differences in SF-36 domains between groups we used the non-parametric Mann-Whitney *U*-test. To analyze the continuous variable age, non-parametric Spearman correlation was used instead and in addition to Pearson correlation as a comparison to confirm results. Age was divided into the following strata: 18-39 years, 40-59 years, and above 60 years. These strata were analyzed using Kruskal-Wallis non-parametric analysis of variance. The magnitude of group differences was further determined by calculation of effect sizes (ESs). ES of a between-group difference was estimated by calculating the mean difference, divided by the pooled standard deviation (Cohen’s *d*). ES was interpreted according to standard criteria: trivial (<0.20), small (0.20-0.49), moderate (0.50-0.79), and large (≥0.80) [13]. Two-sided *p*-values <0.05 were considered statistically significant, whereas associations with *p*-values between 0.05 and 0.10 were considered a tendency. For statistical analyses we used Excel 2010 (Microsoft Corporation, Redmond, WA), SPSS version 22 (IBM, Armonk, NY), and SAS version 9.2 (SAS Institute Inc., Cary, NC).

### Definitions of variables

*Appropriate ICD therapy* was defined as overdrive-pacing or discharge of 30-42 Joules due to a ventricular arrhythmia episode above the programmed detection interval and duration. An ICD shock that is *not* due to ventricular arrhythmia is called *inappropriate* and results from inappropriate detection or interpretation of cardiac potentials (atrial arrhythmias, T-wave oversensing) or signals outside the heart (lead failure, myopotentials, magnetic fields). *Systolic HF* implies symptoms like dyspnea or exercise intolerance and an echocardiogram with an ejection fraction (EF) <50 %. *Surgical complications* are defined as those complications related to the device system or the implant procedure, but not elective replacement due to battery depletion. Risk factors that would have been assessed in primary indication ICD patients are often not systematically addressed in secondary prevention cases, because the patient already has a clear indication. Thus, analyses of risk factor subgroups are limited to primary prevention patients. The risk factors analyzed were *non-sustained ventricular tachycardia (NSVT)*, *unexplained syncope*, and a *family history of SCD* in a first degree relative before the age of 55 years.

### SF-36 domains and validation

Generic HRQL was assessed by the SF-36 health survey version 1 [7]. SF-36 is a patient-reported multidimensional HRQL instrument developed in the Medical Outcome Study and used in thousands of studies since the introduction in the 1990s [6]. It is useful for estimating the perceived burden of different medical conditions. The instrument has 36 items measuring eight domains that reflect a wide spectrum of physical and mental health aspects: physical functioning (PF), role-physical (RP), bodily pain (BP), general health (GH), vitality (VT), social functioning (SF), role-emotional (RE), and mental health (MH). Domain scores range from 0 to 100 with higher values indicating better HRQL. The eight domains can be aggregated into two summary measures: the physical component summary (PCS) score and the mental component summary (MCS) score. PCS and MCS are calculated using norm-based scoring with a mean of 50 and a value above 50 indicating better HRQL than the general Swedish population. In the Swedish validation of SF-36, internal consistency reliability estimates (Cronbach’s α) for the eight domains ranged between 0.79 (role-emotional) and 0.93 (bodily pain) [6].

The SF-36 profile in the study group was compared to a general population sample randomly selected from the Swedish SF-36 normative database (*n* = 8930; response rate 68 %) [7]. The normative sample (validated in Sweden 1991-92) was matched on sex and age and comprised 735 persons (516 males) with a mean age of 55.9 years (SD 14.8).

## Results

### Cohort characteristics

**Fig. 1:**
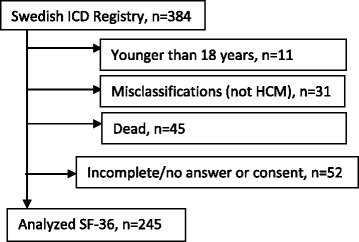
Flow-chart of SF-36 questionnaire inclusion in hypertrophic cardiomyopathy patients with implantable defibrillators

A total of 245 ICD patients with a validated diagnosis of HCM reported their HRQL according to SF-36 (response rate 82.5 %) as depicted in the flow-chart (Fig. 1). Mean age was 55.9 years (SD 14.7, range 19-88 years), and a majority was men (70.2 %). There were no gender differences concerning mean age (men 56.4 years vs women 54.8 years; *t*-test *p* = 0.420). Mean ages were similar with regard to gender in the stratum; 18-39 years (men 29.7 years vs women 31.1 years, *p*-value 0.507), 40-65 years (men 51.1 years vs women 50.6 years, *p*-value 0.692), and older than 65 years (men 68.8 years vs women 67.5 years, *p*-value 0.256). Characteristics of the patients are shown in Table 1. There was no significant difference in any of these variables between primary and secondary indication patients. Among primary prevention patients, 101 (56.1 %) reported a history of non-sustained ventricular tachycardia, 43 (23.9 %) had a family history of SCD, and 62 (34.4 %) had unexplained syncope as a risk factor justifying the decision to implant an ICD. All ICD device systems were implanted transvenously. Moreover, 72 (29.4 %) patients had a complication related to the ICD requiring surgical intervention. Inappropriate ICD shock was experienced by 33 (13.5 %) patients, and was often triggered by atrial arrhythmias. A potentially life-saving, appropriate ICD therapy occurred in 56 patients (22.9 %).

**Table 1:** Characteristics of 245 hypertrophic cardiomyopathy patients with implantable defibrillators

Age, mean (years)	55.9	(SD 14.7)
ᅟ18-39 years	38	15.5 %
ᅟ40-65 years	92	37.6 %
ᅟ≥65 years	115	46.9 %
Male	172	70.2 %
Primary prevention	180	73.5 %
Diabetes mellitus	18	7.3 %
Hypertension	26	10.6 %
Stroke	23	9.4 %
Myocardial infarction	4	1.6 %
Alcohol septal ablation	17	6.9 %
Myectomy	16	6.5 %
Valvular surgery	8	3.3 %
Atrial fibrillation	87	35.7 %
Heart failure	48	19.6 %

### HRQL in the ICD cohort compared to general population norms

**Fig. 2:**
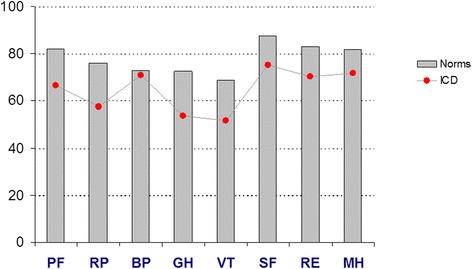
Bar-chart SF-36 score in 245 hypertrophic cardiomyopathy patients with implantable defibrillators compared to Swedish age- and sex matched population norms (*n* = 735)

All SF-36 scales showed a lower HRQL in HCM patients with ICDs compared to sex- and age-matched population norms (Table 2 and Fig. 2). There was a marked statistically significant (*p* < 0.001) difference for all scales except BP (*p* = 0.550). The effect size between the study cohort and the matched population norms with regard to the significant differences varied between 0.35 (RE) and 0.77 (GH). PCS effect size was 0.62 and MCS 0.46, respectively. Thus, effect sizes were small to moderate and only approached large with regard to GH. Men and women reported roughly equal SF-scores but men had a tendency to higher PF scores (*p* = 0.053). Increasing age was associated with lower scores on PF, RP, and PCS, and higher scores on MH and MCS. Age had significant influence (Spearman coefficients) on PF (*r* = -0.33; *p* < 0.001), RP (*r* = -0.18; *p* = 0.003), MH (*r* = 0.13; *p* = 0.038), PCS (*r* = -0.30; *p* < 0.001), and MCS (0.14; *p* = 0.024). Younger patients reported higher scores on the physical scales PF, RP, and PCS, while older patients scored higher on the mental scales MH and MCS. There were significant differences on PF, PCS and MCS among the age strata 18-39 years, 40-59 years, and 60 years.

**Table 2:** SF-36 score in hypertrophic cardiomyopathy patients with implantable defibrillators compared to general Swedish population norms

SF-36 domains	Cohort mean	SD	95 % CI	Effect size	Norm mean	SD	95 % CI	*p*-value
Physical functioning	66.6	27.6	63.1-70.0	0.62	82.1	22.4	80.5-83.8	<0.0001
Role physical	57.4	43.6	52.0-62.9	0.46	76.0	37.1	73.2-78.7	<0.0001
Bodily pain	70.7	29.4	67.0-74.4	0.08	72.9	27.3	70.9-74.9	0.550
General health	53.7	25.5	50.5-56.9	0.77	72.4	23.1	70.7-74.1	<0.0001
Vitality	51.8	26.2	48.5-55.1	0.67	68.7	24.4	66.9-70.5	<0.0001
Social functioning	75.1	26.9	71.7-78.5	0.52	87.7	21.3	86.2-89.3	<0.0001
Role emotional	70.1	40.8	64.9-75.2	0.35	82.9	32.0	80.5-85.3	<0.0001
Mental health	71.8	22.9	69.0-74.7	0.47	81.7	18.8	80.3-83.1	<0.0001
Physical Component Summary	40.8	12.4	39.3-42.4	0.62	47.9	10.5	47.1-48.7	<0.0001
Mental Component Summary	45.5	12.9	43.9-47.1	0.46	50.8	10.2	50.0-51.6	<0.0001

### HRQL subgroup analyses

Patients in the ICD cohort were dichotomized into subgroups according to clinical markers and between-group differences in HRQL were tested. Outcomes showing a significant (*p* < 0.05) or a tendency (*p* < 0.10) towards a significant difference with regard to AF, systolic HF, appropriate ICD therapy, and inappropriate ICD shock are reported in Table 3 along with effect size. Patients with a history of AF reported significantly worse physical health (PF, RP, GH, PCS), worse SF, and a borderline trend towards worse BP. Effect sizes were in the small range, but estimates for PF and PCS were close to the limit for moderate ES. Patients with a history of AF were 7.4 years older (*p* < 0.001) than patients without AF. Systolic HF was also associated with a deteriorated physical health (PF, RP, GH, PCS) and ESs were small to moderate. There was a tendency towards worse VT and SF with small ESs. Mean age was 4.6 years higher in HF patients compared to patients without HF (*p*-value = 0.053).

**Table 3:** Subgroup analyses of hypertrophic cardiomyopathy patients with implantable defibrillators

SF-36 domains	Atrial fibrillation		Heart failure		Appropriate therapy		Inappropriate shock	
	*p*-value	ES	*p*-value	ES	*p*-value	ES	*p*-value	ES
Physical functioning	**<0.001**	0.47	**<0.001**	0.68	0.812		0.119	
Role physical	**0.002**	0.38	**0.003**	0.48	0.211		0.382	
Bodily pain	**0.051**	0.26	0.260		0.229		0.188	
General health	**0.004**	0.38	**0.023**	0.33	0.864		0.118	
Vitality	0.234		**0.075**	0.30	0.166		**0.080**	0.31
Social functioning	**0.004**	0.42	**0.069**	0.36	0.180		**0.058**	0.37
Role emotional	0.234		0.195		0.209		**0.028**	0.42
Mental health	0.288		0.681		**0.033**	0.30^a^	0.242	
Physical Component Summary	**<0.001**	0.48	**<0.001**	0.63	0.735		0.252	
Mental Component Summary	0.495		0.884		**0.076**	0.27^a^	**0.060**	0.38

*ES* effect size

^a^ *higher* mental health and mental health summary scores (all other effect sizes were *lower*)

HCM patients who experienced appropriate ICD therapy had better mental health with a small effect size and MCS showed the same trend. Age was not different between the two categories, regardless of the history of appropriate ICD therapy (*p* = 0.290). Mental health tended to be worse (RE, VT, SF, MCS) among patients who had experienced inappropriate shock(s). There was no age difference between those who had experienced an inappropriate shock or not (*p* = 0.381).

Comparison of patients with secondary versus primary indication showed no significant difference in SF-36 scores, but there was a tendency towards better vitality (*p* = 0.07; ES 0.28) among secondary preventive patients. Subgroup analysis of primary prevention patients with the risk factor NSVT or familial SCD showed no significant difference in the SF-36 domains. Patients with or without a history of syncope had similar SF-36 scores but syncope patients showed a tendency towards worse GH (*p* = 0.07; ES 0.27). Patients who had undergone surgical procedures due to device system complications had similar SF-36 scores compared to other patients.

## Discussion

Hypertrophic cardiomyopathy patients with ICDs report a poor HRQL. The generic instrument SF-36 showed lower levels in all eight domains compared to general Swedish age- and sex-matched population norms. The difference in scores was highly significant (*p* < 0.001) for all domains and their physical and mental component summary components, except bodily pain. Effect sizes varied from small to moderate with the largest value in the GH domain (0.77). Physical component summary was moderately affected (0.62) while MCS was smaller (0.46); thus the effect on HRQL was even more pronounced on physical domains. The results confirm previous findings presented more than 15 years ago from a highly specialized British center. This British study included 137 HCM patients (not an ICD cohort) and showed a deteriorated health status (SF-36) that was similar, or in mental domains even worse, to the severely ill cardiac patients in the Medical Outcome Study [6]. Recently, a study of 19 HCM patients in Norway demonstrated poor HRQL in both physical and mental domains of SF-36 [14]. Another study showed that HCM patients in general have disturbed sleep quality [15], which may have a negative impact on both physical and mental HRQL. The present nationwide study demonstrates a markedly poor HRQL in HCM patients with ICDs, despite modern treatment options including pharmacological optimization, alcohol septal ablation, septum reduction surgery, pacemakers, cardiac resynchronization therapy and treatment of comorbidities [1].

Specific treatment like alcohol septal ablation can reduce gradients and suggests at least short-term improved HRQL [16]. However, no prospective study has so far addressed the long-term effects on HRQL of any interventions in the specific groups of HCM patients with ICDs. Patients with obstructive HCM may experience symptom relief by pacing and subsequent outflow gradient reduction, or by a possible placebo effect. In our study, a total of 33 patients underwent procedures to reduce outflow obstruction, either by alcohol septal ablation or myectomy. Previous studies show that SF-36 scores on both physical and mental domains improved during the first year of pacing treatment [17–19]. HRQL ratings may thus depend on time since ICD implant and might actually reflect individual coping strategies after the decision to implant. In a general ICD population at least one year after ICD implant, HRQL was significantly poorer than the general population and scores on the SF-36 were lower on the physical components than on the mental domains and PCS [20]. Notably, only 3 % of the patients in our study had their first implant during the last 12 months, which implies that the HRQL assessment does not reflect temporary changes due to the implant procedure and the reasons for ICD implant. Although we live in a modern era with advancements in specific HCM treatment and overall cardiac care and comorbidities, the present study shows a poor HRQL in HCM patients with ICDs. These patients often have a reasonable life expectancy compared to other ICD recipients and the association between clinical markers and poor HRQL deserves further attention.

### Atrial fibrillation

The poor HRQL in HCM patients with ICDs may be partly explained by a history of AF. Subgroup comparisons in the present study showed that patients with a history of AF (36 %) scored worse on SF-36 than those without AF. This was especially pronounced with regard to physical domains with significantly lower scores on PF, RP, GH, SF, and PCS. The strongest impact was seen on PF and PCS with a close to moderate effect size of 0.47 and 0.48 respectively. Thus, AF, defined as paroxysmal, persistent, or permanent, was a major determinant of poor physical health in our study. AF is associated with worse HRQL outcome in previous studies of other patient groups and this finding aligns with the present study [21]. The deleterious burden of symptoms due to AF is well known in HCM [1]. AF is also the main cause of inappropriate ICD shocks and contributes to HF. It is therefore important to control rhythm whenever possible or, in permanent AF, to control the rate. Due to the risk of embolization stroke, a history of AF warrants anticoagulation therapy, independent of other risk factors [1]. AF is major cause of worsening symptoms in HCM and requires vast healthcare resources. The patients with AF in this study were 7.4 years older than those without AF, suggesting that more patients will develop AF over an extended follow-up with the likelihood of a concomitantly deteriorated HRQL. Thus, our finding of a strong association between AF and poor HRQL is expected, but we show that AF affects several aspects of health, mental as well as physical, thus adding to our knowledge about the consequences of AF in patients with HCM. This is a major clinical challenge, because a substantial proportion of HCM patients has or will develop AF.

### Heart failure

Patients with HF in the study scored worse on SF-36. A history of HF was associated with significantly lower scores in PF, RP, GH, and PCS and scores with borderline *p*-values on the domains VT and SF. The effect size varied from 0.30 (VT) to 0.68 (PF). Notably, the ES for PF among HF patients was the strongest of all subgroup comparisons in this study. HF patients were older (mean 4.6 years) but this likely has only a slight influence (*t*-test *p*-value = 0.053). HCM patients in general often suffer from dyspnea, attributable to outflow obstruction, disturbed relaxation the left ventricle, arrhythmias, and comorbidities. As expected, the HF group with EF <50 % reported poorer HRQL, especially with regard to physical domains. The SF-36 physical domains showed higher ES-values and significance levels, which may be explained by the impact of the patient’s deteriorating clinical course of worsening HF. An EF <50 % in a HCM patient is often a sign of poor overall prognosis. Our findings are in accordance with experience from other patients with HF. Indeed, in stable, chronic, symptomatic HF patients, all SF-36 domains were affected, which parallels findings among ICD recipients with HF [22–24]. In our study, the 20 % of patients with HF defined as EF <50 % turned out to have worse physical health. Furthermore, exertional dyspnea is common among all HCM patients and may be an explanatory variable to the overall poor HRQL. Due to the higher impact on physical domains in this study, physical limitations should be evaluated thoroughly in HCM patients along with their ICD device follow-ups.

### Appropriate therapy and inappropriate shocks

Patients who had at least one appropriate therapy reported significantly better mental health than those without a potentially life-threatening ventricular arrhythmia terminated by the ICD. The Mental component summary showed a tendency in the same direction. This finding comes as a relief, because events necessitating ICD therapy can provoke anxiety or other concerns in the patient (for example driving restriction) and in the family. On the other hand, knowing that ICD therapy may have aborted a potentially lethal arrhythmia may have a positive psychological impact on the patient (gratitude, relief, sense of safety).

Inappropriate shocks also affected mental health in a negative way. They were associated with a significantly lower RE score, while VT, SF and MCS showed the same tendency. The effect sizes were small and this supports the notion that many patients eventually learn to cope with inappropriate shocks. Nevertheless, avoiding inappropriate shocks remains important as inappropriate therapy may undermine ICD acceptance in both patients and healthcare providers.

This cohort reported a rate of 5.3 % per year appropriate therapy and 4.0 % per year inappropriate shocks, which is within the range of the pooled data in the review study [3, 12]. This suggests that results of the present study are generalizable, even though none of the studies in the review included HRQL assessment.

### Primary versus secondary ICD indication

In this study there were no significant differences between patients based on device indication but there was a tendency towards better vitality among survivors after cardiac arrest or ventricular tachycardia with hemodynamic compromise. Prophylactic ICD patients may cope differently with the implant decision than secondary prevention patients. The latter group may feel gratitude because they survived a life-threatening event, but they may also experience anxiety and depression. In another study of general ICD recipients, there were lower SF-36 scores in primary than in secondary prevention patients (significantly lower in all domains except BP) [25]. This underscores the importance of being aware of the vulnerability among primary prevention patients, including HCM patients. HRQL aspects should be integrated in the overall evaluation of the patient and in the decision-making process, first when offering a potential candidate an ICD but also subsequently during follow-up. These strategies may include support groups where patients can share experiences under guidance of a healthcare provider, qualified coaching in coping strategies, individual counselling, and optimized management [26].

Interestingly, a French study on ICD recipients with Brugada syndrome and similar age- and sex distribution as patients in our present study sample showed no difference on BP and SF compared to the general population [27]. Furthermore, a Dutch study confirmed that symptomatic HCM was a determinant of lower scores on the SF-36 physical component. However, mutation carriers without manifest disease reported excellent HRQL, even better than the general population [26, 28]. This highlights that the burden of symptoms are the crucial determinants of poor HRQL in HCM patients [29, 30].

There is convincing support from our study that poor HRQL in HCM patients with ICDs can be mainly attributed to the burden of symptoms of the underlying disease and its different manifestations.

### Familial SCD, non-sustained ventricular tachycardia, and syncope

In our analyses of primary prevention patients we found no specific impact on HRQL in the group of patients who had a first-degree family member with SCD. Neither a history of NSVT nor unexplained syncope seems to affect HRQL. It is also reassuring that patients who underwent surgical procedures related to ICD device system complications had similar HRQL as other patients, which suggests that, as a group, they cope well with these interventions in the long term.

### Sex differences

In the present study, no significant sex differences in SF-36 scores were observed, although PF tended to be slightly better in men. Likewise, another large study of general ICD patients confirmed lower PF scores in females but also lower VT scores, while no other differences in SF-36 were significant. In the same study, appropriate ICD therapy was only associated with anxiety and lower GH (*p* = 0.03) but inappropriate shock was not related to any SF-36 domain [31]. This study supports the concept that comorbidities, like AF and HF, rather than sex determine poor HRQL. The decision to implant an ICD should be based on individual risk and benefit estimation rather than gender category.

### Clinical perspectives

This study supports previous findings showing that the major determinants of HRQL are related to the burden of symptoms among HCM patients but not related to sex. The decision to implant an ICD should take HRQL aspects into consideration and include comorbidities as a crucial determinant of poor HRQL. However, complications of ICD therapy do not have a significant impact on HRQL and should not be considered a reason to avoid an ICD implant. It is important to provide eligible patients with thorough information before consent and also to address potential ICD-related concerns during follow-up, which may involve many years. ICD therapy should include tailored device programming in order to avoid inappropriate shocks and also psychological support [32, 33]. Concerning quality of life improvement, overall management of HCM and comorbidities are important but remains challenging despite advances in many therapeutic fields.

### Strengths and weaknesses

This is the largest study on generic HRQL in HCM patients with ICDs. It is nationwide study of unselected patients using the well-validated SF-36 instrument with age- and sex-matched comparisons with general population norms. The response rate in the study was high and register data were validated using medical records. Assessment of HRQL was done after the initial period of ICD implant and is thus likely to reflect a more stable phase for the patient. However, the cross-sectional design does not deal with the variation in HRQL over time that is likely to occur in these patients, who often have long clinical courses. In addition, HRQL is self-reported and patients may have concerns that are not covered in a generic health assessment questionnaire such as the SF-36. Even though SF-36 is well validated, analyses on associations with clinical variables lack definite causative inference and are just associations.

## Conclusions

Hypertrophic cardiomyopathy patients with ICDs have poor HRQL compared to the general population. AF and HF were major determinants of poor perceived health, especially physical health status. Appropriate ICD therapy for ventricular arrhythmias implies similar HRQL scores, while patients who receive inappropriate shocks report lower mental health. There were no significant sex differences. In order to improve HRQL, HCM and its comorbidities must be better managed, ICD issues must be carefully addressed, and patients should receive support to promote their general well-being.

## Abbreviations

AF: atrial fibrillation
BP: bodily pain
CI: confidence interval
EF: ejection fraction
GH: general health
HCM: hypertrophic cardiomyopathy
HF: heart failure
HRQL: health related quality of life
ICD: implantable cardioverter defibrillator
MH: mental health
NSVT: non-sustained ventricular tachycardia
PF: physical functioning
RE: role emotional
RP: role physical
SCD: sudden cardiac death
SD: standard deviation
SF: social functioning
SF-36: short form 36
VT: vitality
